# Targeting BRAF for patients with melanoma

**DOI:** 10.1038/sj.bjc.6606030

**Published:** 2010-12-07

**Authors:** H-T Arkenau, R Kefford, G V Long

**Affiliations:** 1 Sarah Cannon Research UK, 93 Harley Street, London W1G 6AD, UK; 2 Melanoma Institute Australia and Westmead Hospital, University of Sydney, New South Wales, Australia

**Keywords:** BRAF, BRAF^V600E^, melanoma, PI3K-AKT-mTOR, RAS-RAF-MEK-ERK

## Abstract

The prognosis of patients with metastatic melanoma is poor and not influenced by systemic therapy with cytotoxic drugs. New targeted agents directed against the RAS-RAF-MEK-ERK pathway show promising activity in early clinical development and particular interest is focused on selective inhibitors of mutant BRAF, which is present in one half of the cases of metastatic melanoma. The majority of patients on early trials of these drugs develop secondary resistance and subsequent disease progression and it is, therefore, critical to understand the underlying escape mechanisms leading to resistance.

## Melanoma: a heterogeneous disease

Metastases develop in 10–15% of patients with cutaneous melanoma and there is no evidence from phase-III trials that systemic treatment prolongs survival, nor is there an effective adjuvant therapy after resection of high risk Stage I–III disease (Thompson *et al*, 2005). Only 5% of patients with visceral metastases survive for 2 years (Balch *et al*, 2001).

**Figure 1:**
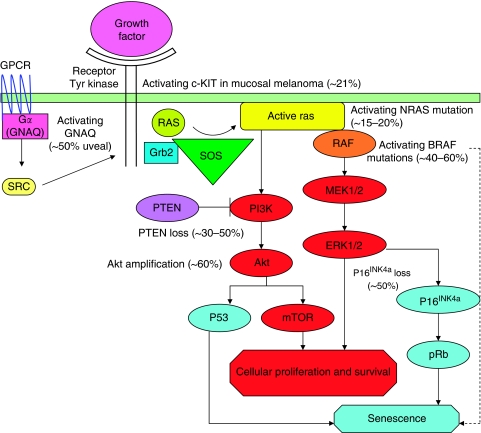
The MAPK pathway activation in melanoma. Oncogenic NRAS, BRAF, GNAQ and CKIT signal through the MAPK pathway. Oncogenic NRAS also induces the phosphatidylinositol-3′ kinase (PI3K) cascade. MAPK signalling can lead to proliferation in transformed cells, but also induces a potent form of growth arrest, known as senescence in normal melanocytes. The approximate proportion of melanomas with mutations are shown. GPCR=G-protein coupled receptor.

Understanding the genetic heterogeneity in melanoma has become increasingly important with the development of therapies aimed at targeting specific genetic aberrations. Of particular importance in melanoma is the mitogen-activated protein kinase (MAPK) pathway, which normally regulates cell growth, proliferation and differentiation (Figure 1). Aberrant activation of the MAPK pathway is present in over 80% of primary melanomas (Platz *et al*, 2008), and mutations in proteins along the RAS-RAF-MEK-ERK pathway are thought to be mutually exclusive. Such mutations have been documented in all subtypes of melanoma (Curtin *et al*, 2005), including cutaneous (50–60% BRAF, 15% NRAS and 17% CKIT chronic sun damage), mucosal (11% BRAF, 5% NRAS and 21% CKIT) and uveal (50% GNAQ) melanomas. The BRAF and NRAS mutations have not been reported in uveal melanoma to date (Populo *et al*, 2010).

## Types of BRAF mutations

**Figure 2:**
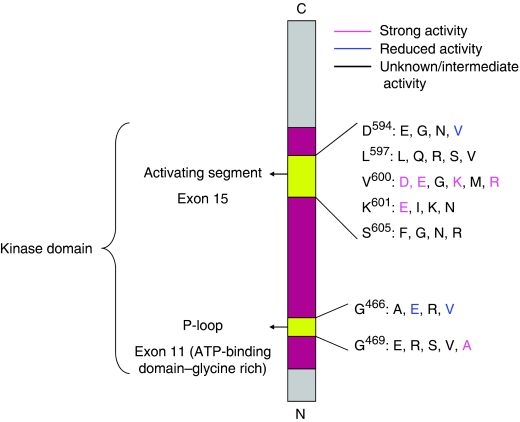
Common types of BRAF mutations in melanoma (Wan *et al*, 2004).

Constitutively activating somatic missense mutations in BRAF were discovered to be present in a wide variety of human cancers, including papillary thyroid cancer (39–69%), cholangiocarcinoma (22%), colorectal cancer (5–12%) and borderline ovarian cancer (30%) (Catalogue of Somatic Mutations in Cancer (COSMIC) website). The mutations are either in the activating segment in exon 15 or the glycine-rich loop (P-loop) in exon 11 of the kinase domain of the BRAF protein (Figure 2). The point mutation in DNA (1799T → A) resulting in a single amino-acid substitution at Valine 600 to Glutamic acid in the activating segment (V600E, previously known as V599E) is the most common change, and is found in 80% of the mutated tumours (Davies *et al*, 2002). This is distinct from the CC → TT or C → T changes associated with ultraviolet light exposure in non-melanoma skin cancers. BRAF V600E results in elevated kinase activity compared with BRAF wild type and stimulated phosphorylation of downstream endogenous ERK (Dhomen and Marais, 2009).

Today more than 75 somatic mutations in the BRAF gene have been identified in melanoma, and all mutations at V600 in Exon 15 constitutively activate BRAF (Figure 2). In BRAF mutant melanoma, 74–90% are V600E (Platz *et al*, 2008) and 16–29% are V600K mutations (Thomas, 2006; Long *et al*, 2010). One hypothesis for the mechanism of uncontrolled activation is the increased exposure of the activation segment for interaction when a small hydrophobic amino acid at 600 (Valine) is switched to a hydrophilic residue. Normally, the RAF kinase domain in the inactive conformation is hidden in a hydrophobic pocket (Wan *et al*, 2004). A small number of mutations have reduced kinase activity compared with wild type, for example, G465E, G465V, D593V and G595R, but cause increased ERK activation, possibly via binding and activation of CRAF (Houben *et al*, 2004; Wan *et al*, 2004).

## Prognosis of BRAF mutation in metastatic melanoma

The frequency of BRAF mutation in primary melanomas ranges from 36 to 45% (Curtin *et al*, 2005; Liu *et al*, 2007; Thomas *et al*, 2007) and 42–55% in metastatic melanoma (Houben *et al*, 2004; Ugurel *et al*, 2007; Long *et al*, 2010). The presence of a BRAF mutation in early melanoma shows no association with disease-free interval or overall survival (Shinozaki *et al*, 2004). In contrast, the presence of a BRAF mutation in metastatic melanoma is associated with a poorer survival from time of first metastasis (Long *et al*, 2010) or time from first resected metastasis (Houben *et al*, 2004), although not consistently observed in smaller studies (Ugurel *et al*, 2007).

## Rationale for BRAF inhibition in melanoma

Advanced melanomas often have multiple genetic defects affecting diverse biochemical pathways. It was, therefore, surprising that those with activating BRAF mutations displayed the hallmarks of oncogene addiction. When this single oncogenic alteration was targeted in melanoma cell lines with specific inhibitory nucleic acids or chemical RAF inhibitors, the cell lines displayed growth arrest and induction of apoptosis (Calipel *et al*, 2003). The behaviour of mutant BRAF melanomas in murine xenograft models supported these preclinical findings and confirmed mutant BRAF as an attractive target for melanoma therapy, particularly as it occurs in at least half the tumour population, and does not occur in normal cells. In addition, its serine/threonine kinase domain was amenable to the rational design of selective inhibitors.

## Early clinical data of RAF inhibitors in melanoma patients

The first RAF kinase inhibitor entering early clinical trials was the oral diphenyl urea, sorafenib (Bay 43-9006). Sorafenib has potent RAF isoform kinase inhibitor activities (CRAF (IC_50_ 6 nM)>wild type (wt) BRAF (IC_50_ 22 nM)>mutant BRAF^V599E^ (IC_50_ 38 nM)), but was also found to have a much broader inhibitory profile, including kinases of the vascular endothelial growth factor receptor 2 and 3 (VEGFR-2 (IC_50_ 90 nM) and VEGFR-3 (IC_50_ 20 nM)), the platelet-derived growth factor receptor-*β*(IC_50_ 57 nM), Flt-3 (IC_50_ 58 nM) and c-Kit (IC_50_ 68 nM).

In human xenograft models sorafenib resulted in prolonged growth stabilisation rather than tumour response, perhaps forewarning the negligible clinical activity in melanoma patients who were treated within a discontinuation phase-II trial (Karasarides *et al*, 2004; Eisen *et al*, 2006). These results raised questions about the *in human* BRAF inhibitory activity of sorafenib and raised scepticism about the relevance of mutant BRAF as a target in melanoma. Activity of the drug in renal cell and hepatocellular carcinoma has since been attributed to promiscuous inhibitory effects on receptor tyrosine kinases, including VEGFR, PDGFR and cKIT.

Further research led to the development of second generation more selective RAF inhibitors, which are currently in clinical trials (Table 1).

XL281 (famotidine) is an orally administered inhibitor of the wt BRAF (IC_50_ 4.5 nM), CRAF (IC_50_ 2.5 nM) and mutant BRAF^V600E^ (IC_50_ 6 nM) kinases and demonstrated potent inhibitory effect in a variety of human xenograft models. The phase-I study of XL281 (NCT00451880) in an unselected patient population showed prolonged disease stabilisation in two patients with BRAF^V600E^ mutant papillary thyroid cancer (>15 and 17 months, respectively) and some minor responses in nine patients with colorectal, ovarian and non-small-cell lung cancer. Overall XL281 was well tolerated with acceptable rates of grade 3/4 nausea, vomiting and diarrhoea (Schwartz *et al*, 2009). Currently, a three-arm dose expansion part is expected to recruit a total of 180 patients, including patients with melanoma, papillary thyroid and colorectal and non-small-cell lung cancer. Results of an ongoing melanoma phase-I/II trial (RAF265-MEL01; wt BRAF and BRAF mutant patients included) are awaited.

PLX4032 (RO 5185426) is an ATP competitive, orally administered BRAF inhibitor (wt BRAF IC_50_ 100 nM) with high selectivity for the mutant allele (BRAF^V600E^ IC_50_ 31 nM) and has shown selective tumour suppression in mutant BRAF cell lines and xenograft models. In a phase-I clinical trial enriched for patients with mutant BRAF metastatic melanoma 11/16 (68%) achieved partial response (PR) and four patients had minor responses leading to a progression-free survival (PFS) of 8–9 months (Flaherty *et al*, 2009). The dose expansion cohort enroled a selected group of BRAF^V600E^ melanoma patients (*n*=32) at the MTD dose level of 960 mg twice daily (Chapman *et al*, 2009). In this group, a total of 26 patients (81%) had response (two CR and 24 PR). Overall PLX4032 was well tolerated with mild nausea and vomiting, skin rash and diarrhoea, but 21% of the patients on active doses developed cutaneous neoplasms, including keratoacanthoma (KA), low-grade squamous cell carcinoma (SCC) and verrucal-like lesions. These lesions occurred within 8–12 weeks from treatment start and were resectable (Flaherty *et al*, 2010a, 2010b). On the basis of these promising results, a phase-II trial (BRIM 2, NCT00949702) is now fully accrued and a phase-III trial (BRIM3, NCT01006980) comparing PLX4032 to standard chemotherapy with the alkylating agent dacarbazine in untreated patients with BRAF^V600E^ mutant metastatic melanoma is underway.

GSK 2118436 is an ATP competitive, reversible inhibitor of the mutant BRAF^V600E/K/D^ (IC_50_ 0.5, 0.6, 1.9 nM, respectively), wt BRAF (IC_50_ 12 nM) and CRAF (IC_50_ 5 nM) kinases with promising preclinical efficacy data in melanoma. A phase-I trial (NCT00880321) of this oral compound enroled >100 patients with BRAF mutations (primarily melanoma patients) (Kefford *et al*, 2010). Overall GSK 2118436 showed good tolerability with grade 1/2 nausea, fatigue, fever, headaches and skin rash as the main side effects. In total, 9% of the patients developed cutaneous neoplasms, including low-grade SCC that occurred between weeks 2 and 14. Preliminary pharmacodynamic data showed dose-dependent phospho-ERK inhibition and a correlation with clinical response. Clinical responses (63% PR) were seen at the recommended phase-II dose (150 mg twice daily), with responses in lung, liver, bone and brain metastases. Importantly, these responses were not only seen in patients with BRAF^V600E^ but also in BRAF^V600K^ and BRAF^V600G^ mutations, and possibly shows the wider range of activity of selective BRAF inhibitors that are not limited only to BRAF^V600E^-mutant tumours. These findings were also supported by a recent case report of the BRAF inhibitor PLX4032 in a BRAF^V600K^ mutant patient (Rubinstein *et al*, 2010).

A phase-II study of GSK 2118436 is currently underway as salvage therapy in mutant BRAF metastatic melanoma and a phase-I study has commenced of GSK 2118436 in combination with the MEK-inhibitor, GSK1120212, (NCT01072175) in BRAF mutant melanoma patients, a strategy of tandem MAPK inhibition.

Other selective B-RAF inhibitors are currently in preclinical and early clinical development and include GDC-0879, ARQ736 and AZ628, among others.

## Specific side effects of selective RAF inhibitors

Selective RAF inhibitors display good tolerability with infrequent severe toxicities. Among the common grade 1–2 adverse events are skin changes (50–70%), fatigue (30–50%), diarrhoea (10–30%) and nausea (10–20%). In the GSK 2118436 phase-I trial, 29% of patients reported mild grade 1/2 headaches and a possible cytokine-related fever in 37% of the patients.

Most concerning was the development of cutaneous lesions, including KA and SCC in 15–30% of patients on the GSK 2118436 and the PLX4032 studies (Flaherty *et al*, 2010a, 2010b; Kefford *et al*, 2010). The biology and natural history of these lesions, in comparison to their spontaneouas counterparts is unknown, and no systemic spread has been reported to date. One possibility to explain the underlying mechanism could be an exacerbation of upstream RAS mutations in pre-existing skin lesions. RAS mutations occur in approximately 15% of SCCs and, under these conditions, alterations in downstream dimerisation of BRAF by selective BRAF inhibitors could lead to paradoxical activation of the MAPK pathway in squamous cells.

Notably, the phenomenon of paradoxical activation of the MAP kinase pathway has been reported with mutant-selective BRAF inhibitors under certain conditions (Hatzivassiliou *et al*, 2010; Heidorn *et al*, 2010; Poulikakos *et al*, 2010). Although inhibition of MEK and ERK phosphorylation was achieved in BRAF^V600E/K^ mutant melanoma, significantly increased tumour growth was seen in BRAF wild-type melanomas with upstream RAS mutations. It is proposed that in BRAF wild-type tumours BRAF signalling is usually inactivated via BRAF-induced autophosphorylation, but this self-inhibitory process is interrupted by selective BRAF inhibition. Subsequently, in a RAS-dependent manner BRAF is recruited to the plasma membrane and hyper-activates CRAF, which in turn signals downstream to MEK and ERK. This phenomenon was not observed with non-selective RAF inhibitors, probably because of the promiscuous pan-RAF inhibitory effects. Similar mechanisms are thought to underlie the induction of pre-malignant and malignant changes in keratinocytes. Some of the skin changes seen in patients on selective RAF inhibitors mimic those seen in patients with hereditary gain-of-function mutations in the MAP kinase pathway (Roberts *et al*, 2006), and the frequent induction of SCCs may be due to the same process in keratinocytes carrying RAS mutations.

These findings have important clinical implications, and highlight the need for genotyping tumours before treatment with selective RAF inhibitors and screening for secondary tumours.

## Mechanism of resistance

Similar to other kinase inhibitors (i.e., erlotinib and imatinib), selective RAF inhibitors can lead to drastic and impressive early tumour responses in humans, which may be of short duration in some patients. Approximately 20% of patients with mutant BRAF melanoma show no response, and most patients relapse, with a median PFS of 8–9 months.

In preclinical studies, a subset of resistant BRAF^V600E^ mutant cell lines demonstrated increased CRAF signalling via BRAF/CRAF heterodimerisation and resulted in a shift from B-RAF to CRAF dependency. In addition post-transcriptional elevation of CRAF protein levels are thought to decrease intracellular bioavailability of the selective RAF inhibitor AZ628 and subsequent development of resistance (Montagut *et al*, 2008).

Amplification of the *CCND1* gene resulting in cyclin D1 over-expression has been reported to be present in 17% of mutant BRAF^V600E^ melanomas with independent stimulatory effects on cell-cycle progression via CDK4 (Smalley *et al*, 2008). Furthermore, point mutations of the downstream kinase isoform MEK1 (P124L, P124S and Q56P), have resulted in changes of the allosteric drug-binding pocket within helices A and C leading to sub-optimal drug binding of the selective RAF inhibitor PLX4720 in mutant melanoma cells in cell culture and *in vivo* (Emery *et al*, 2009).

## Relevance of the AKT pathway

Preclinical studies have demonstrated a close interconnection of the RAS-RAF-MEK-ERK and the PI3K-AKT-mTOR signalling pathways, with complex inter-related feedback loops. For example, NRAS is mutated in about 15% of melanoma and can activate both the signalling pathways. Although BRAF and NRAS mutations are mutually exclusive in the majority of melanomas, dual pathway signalling is also frequently seen in melanoma through functional loss (deletion, silencing and/or mutation) of the tumour suppressor gene *PTEN* (Figure 1). Both, genetic changes are seen in approximately 20% of melanomas (Dankort *et al*, 2009). As a major regulator of the PI3K-AKT axis PTEN loss leads to activation of the AKT/mTOR pathway and, via feedback loops, to phosphorylation of MEK and ERK (Tsao *et al*, 2004).

AKT has a central role in regulating apoptosis and over-expression (via amplification or mutation) of the isoform AKT3 correlates with tumour progression. Recent preclinical studies have shown that inhibitors of PI3K and AKT3 increased apoptosis and stimulated tumour regression (Cheung *et al*, 2008). In BRAF^V600E^ mutant cells, AKT activation was required for melanoma initiation, demonstrating the inter-dependence of these two pathways in melanoma.

Downstream of AKT, increased signalling via mTOR regulates translation of pro-proliferative proteins. In preclinical studies, the mTOR inhibitor temsirolimus reversed these effects, however, this was not reproducible in clinical melanoma trials (Margolin *et al*, 2005). These findings could be in part explained by the dual signalling complex of mTOR, including TORC 1 and TORC2. Although temsirolimus (and other rapalogs) inhibits mTOR via TORC1, the uninhibited TORC2 complex continues to stimulate AKT through phosphorylation (Feldman and Shokat, 2011). Trials of dual TORC1 and TORC2 inhibitors are currently in phase-I studies to inhibit both AKT and mTOR signalling.

## Rationally designed combination drug therapy

There is increasing evidence that combination therapies targeting the RAS-RAF-MEK-ERK and the PI3K-AKT-mTOR may be more effective than single-agent therapies. For example in three-dimensional cell cultures of BRAF mutant melanoma the combination of BRAF and AKT3 directed siRNAs demonstrated significantly higher reduction of tumour growth compared with weak growth inhibition by single-agent administration (Cheung *et al*, 2008). These findings were confirmed in a melanoma xenograft model (Bedogni *et al*, 2006). There is evidence of synergism when MEK and PI3K inhibitors are combined and increased apoptotic activity was also demonstrated with a combination of the mTOR inhibitor rapamycin and sorafenib or an MEK inhibitor (Lasithiotakis *et al*, 2008). In contrast to single-agent activity, these combinations resulted in complete downregulation of the anti-apoptotic proteins Bcl-2 and Mcl-1.

Preclinical studies have also shown a synergism between BRAF and MEK inhibitors, with significantly increased apoptosis and prolonged phospho-ERK inhibition compared with BRAF inhibition alone (Paraiso *et al*, 2010). This hypothesis is currently tested in two phase-I studies. The study, NCT01072175, combines the selective RAF inhibitor, GSK 2118436, and MEK inhibitor, GSK1120212, in patients with BRAF mutant metastatic melanoma and the study, NCT01037127, explores the efficacy of the MEK inhibitor, GSK1120212, in patients with BRAF mutant tumours who previously failed a selective BRAF inhibitor. The design of these trials rests on the observation that MEK activation persists in melanoma cell lines that develop resistance to BRAF inhibition (Montagut *et al*, 2008; Smalley *et al*, 2008).

Currently, clinical trials of selective RAF inhibitors in combination with other kinase inhibitors, such as MEK, mTOR, PI3K or AKT are underway or planned. Issues related to these combinations include overlapping or synergistic toxicities and mechanisms of resistance.

## Combinations of RAF inhibitors with chemotherapy

Although the combination of sorafenib and dacarbazine resulted in 24% response rates compared with 12% with dacarbazine alone, there was no effect on the primary endpoint of PFS (McDermott *et al*, 2008). Two large phase-III trials of sorafenib in combination with carboplatin/paclitaxel in chemotherapy-naive (Flaherty *et al*, 2010a, 2010b) and pre-treated (Hauschild *et al*, 2009) patients with BRAF undefined metastatic melanoma did not meet the primary endpoint of improved overall survival.

Whether combinations of selective RAF inhibitors in patients with BRAF mutant melanoma will result in better outcomes remains to be investigated.

Moreover, there is compelling evidence to combine chemotherapy with other inhibitors of the RAS-RAF-MEK-ERK and PI3K-AKT-mTOR pathways.

For example, preclinical data suggest that taxane resistance may be due to increased MEK signalling resulting in anti-apoptotic changes (Haass *et al*, 2009). On the basis of these results a phase-II trial of a taxane-based chemotherapy plus the MEK inhibitor AZD 6244 is in preparation and patients will be randomised according to their BRAF/NRAS mutational status.

The commonly used chemotherapy agents, cisplatin and temozolomide, have the potential to trigger pro-survival and anti-apoptotic effects by activating the AKT signalling pathway (Sinnberg *et al*, 2009). Interestingly, AKT inhibitors reverse these effects in preclinical studies by reducing the anti-apoptotic proteins Bcl-2 and Mcl-1. On this basis, clinical trials of combinations of PI3K/AKT/mTOR inhibitors with temozolomide or cisplatin are underway.

## Other combinations of selective RAF inhibitors

Heat-shock protein 90 (HSP90) is a molecular chaperone responsible for degradation, stabilisation and activation of a variety of proteins, including HER2, CRAF, BRAF, AKT and MET. In melanoma, HSP90 stabilises CRAF and ARAF and is required for the activity of BRAF^V600E^. In patients with mutant BRAF melanoma a combination of a selective RAF inhibitor with an HSP90 inhibitor may, therefore, inhibit multiple pathways involved in resistance. This rationale is supported by preclinical data of the HSP90 inhibitor geldanamycin (Banerji, 2009), which promotes the degradation of CRAF, a protein known to be commonly over-expressed in resistant BRAF mutant cells.

Epigenetic changes via DNA hyper-methylation or changes in DNA remodelling have an important role in cancer development. For example, epigenetic silencing is a frequent mechanism of functional loss of the tumour suppressor gene PTEN in melanoma. Agents, such as the histone deacetylating inhibitors (HDACIs), may restore the tumour supressor function by reversing the effects of epigenetic silencing (Kuwajima *et al*, 2007). Current single-agent HDACI trials show promising results in melanoma patients (Ryan *et al*, 2005) and combinations with selective BRAF inhibitors are planned.

## Conclusion

Clinical trials of new selective RAF inhibitors are currently recruiting, and two recent ‘proof of concept’ phase-I studies have shown activity in metastatic melanoma, a disease notoriously resistant to systemic treatment.

Despite this progress there are important questions to be addressed in current and planned clinical trials. These include the impact of selective BRAF inhibitors on overall survival and quality of life, the identification of biomarkers of early resistance and relapse, the selection and scheduling of drugs to combine with BRAF inhibitors, and the mechanism and prevention of adverse events, such as SCC.

## Figures and Tables

**Table 1:** Clinical development of RAF kinase inhibitors in patients with advanced melanoma

**RAF inhibitor**	**Chemical class**	**Target**	**Additional target**	**Status**	***n***	**Clinical outcome**	**Toxicity profile grade ⩾2**
*Non-selective RAF inhibitors*							
Sorafenib	Diphenyl urea	CRAF, BRAFwt, BRAF^V599E^	VEGFR-2&3, PDGFR-*β*, FGFR-1, Flt-3, c-Kit	Phase-II	37	SD 19%	Diarhea, HFS
+ Temsirolimus				Phase-I/II	69		Diarrhea, rash Hyperlipidemia
+/− Dacarbazine				Phase-II	101	Improved PFS=NS, OS=NS	Haemtox, nausea, hypertension, bleeding lipase
+/ Carboplatin/paclitaxel				Phase-III (first-line)	270	PFS=NS	Haemtox, nausea, neuropathy, rash, HFS
+/− Carboplatin/paclitaxel				Phase-III (second-line)	823	OS=NS	Haemtox, nausea, neuropathy, rash, HFS
RAF265	Benzazole	CRAF, BRAFwt, BRAF^V600E^	VEGFR-2	Phase-I/II (ST)	211	Recruiting	
							
*Selective RAF inhibitors*							
PLX4032	Pyrrolo, pyridine	BRAF^V600E^, BRAFwt, CRAF		Phase-I (ST)		RR 58%^a^ (PFS 9 months^a^)	Fatigue, rash, arthralgia, SCC
				Phase-II	100	Results awaited	
				Phase-III	680	Recruiting	
GSK 2118436	Thiazole	BRAF^V600E,K,D^ CRAF BRAFwt		Phase-I/II (ST)	100	RR 63%^a^	Nausea, Fatigue Headache Fever Rash SCC
				Phase-III		Planned	
+ GSK 1120212 (MEK-I)				Phase-I	93	Recruiting	
XL281		CRAF, BRAF wt, BRAF^V600E^		Phase-I (ST)	180	Results awaited	Fatigue, N/V Diarrhea

Abbreviations: HFS=hand-foot syndrome; NS=not significant; OS=overall survival; PFS=progression-free survival; RR=response rate; SCC=squamous cell carcinoma; SD=stable disease; ST=solid tumour.

a At the recommended phase-II dose level in melanoma subgroup
